# Sex-Specific Differences in Nutrient Intake in Late Preterm Infants

**DOI:** 10.3390/children11030265

**Published:** 2024-02-20

**Authors:** Pradeep Alur, Sumana Ramarao, Addie Hitt, Simmy Vig, Radha Alur, Naveed Hussain

**Affiliations:** 1 Penn State Health, Hampden Medical Center, Enola, PA 17025, USA; 2 Department of Pediatrics, University of Mississippi Medical Center, Jackson, MS 39216, USA; sramarao@umc.edu (S.R.); svig@umc.edu (S.V.); 3 Connecticut Children’s, Hartford, CT 06106, USA; hussain@uchc.edu; 4 Department of Pediatrics, University of Connecticut School of Medicine, Farmington, CT 06032, USA

**Keywords:** late preterm, sex differences, oral intake, nutrient, calories

## Abstract

Challenging the assumption of uniform nutritional needs in preterm feeding, this study identifies crucial sex-specific disparities in formula milk intake and growth among late preterm infants. Premature infants have difficulty regulating their oral intake during feeds, which is why clinicians prescribe feeding volume, calories, and protein via the nasogastric route. However, premature male and female infants have different body compositions at birth, and, subsequently, there is no evidence to suggest that male and female preterm infants differ in their nutritional consumption once they begin feeding ad libitum. This study investigates whether there are any differences in the volume and nutrient intake between the sexes when fed formula ad libitum. Methods: The study involved a retrospective analysis of preterm infants admitted to the NICU and evaluated between 34 0/7 and 36 6/7 weeks of corrected gestation. Late preterm infants appropriate for gestational age who were spontaneously fed formula milk ad-lib and free of any respiratory support for at least two days were included. The study excluded infants with short gut syndrome, severe chromosomal anomalies, or congenital heart conditions. We included 85 male and 85 female infants in this study. The data collected included sex, gestational age, birth weight, anthropometric data at birth, maternal data, nutritional intake, and neonatal morbidity. Results: This study found that female infants consumed more volume, protein, and calories than male infants. The mean formula intake in female and male infants was 145.5 ± 20.8 mL/kg/d and 135.3 ± 19.3 mL/kg/d, respectively, with *p* = 0.002. However, ad-lib feeding duration was not different between the sexes. Growth velocity was also higher in female infants. Conclusions: This study is the first to demonstrate differences in formula milk intake among late preterm infants fed ad libitum. Additional research is needed to confirm our findings and understand sex-specific differences in neonatal nutrition in extremely early preterm infants.

## 1. Introduction

Preterm male and female infants exhibit distinct body compositions from birth, reflected in sex-specific growth charts, owing to their differing growth rates [1]. For example, at 24 weeks of gestation, male preterm infants tended to weigh more than female preterm infants. The 50th percentile for weight at 24 weeks of gestation for males is 651 g, whereas for females, it is 606 g. In addition, male preterm infants have a higher head circumference and length, which strongly suggests a higher growth rate in males than females. This raises the question of whether the nutritional needs of each sex vary, given the discrepancies in their growth characteristics and body composition. Our study investigated whether nutritional intake differs between male and female late preterm infants.

Preterm infants (any infants born ≤36 weeks gestation) when they are born ≤32 weeks of gestation are developmentally incapable of taking adequate oral feeds due to immature sucking and swallowing coordination. For such preterm infants, the American Academy of Pediatrics (AAP) has proposed guidelines for the initiation of intravenous (IV) parenteral nutrition as soon as possible after birth and to start early oro/nasogastric enteral feeds in small volumes, gradually advancing to 150–160 mL/kg/d as parenteral nutrition is being weaned [2]. Oral feeds are attempted once the infant reaches around 32–33 weeks of corrected gestational age and when the infant is considered developmentally ready (demonstrates feeding cues). In infants that require significant respiratory support, oral feeding may not be feasible even at this gestational age. They may require prolonged nasogastric enteral feeding to maintain the prescribed intake volume, calories, and protein for optimal weight gain [3]. Although male and female infants have different growth trajectories, no distinction is made in the Neonatal intensive care unit (NICU) to determine optimal nutrition requirements between the sexes. The current nutrition guidelines for preterm infants in the NICU are unisex.

Studies have shown that growth velocity and body composition vary between sexes, even in preterm infants. The ‘Intergrowth-21’ study found that, in both preterm and term infants, girls had a higher percentage of fat mass at birth than boys [1]. However, preterm infants cannot regulate their enteral feed intake due to poor oral feeding ability, which poses a challenge. Clinicians deliver calories and protein via the oro/nasogastric route without considering the infants’ sex. This unisex feeding strategy leads to the loss of the normal difference in fat mass percentage between the sexes when preterm infants reach a term-equivalent age and may lead to increased abdominal adiposity [4,5]. It is also evident from a recent individual patient data meta-analysis on infants born early or small that, in toddlers, supplementation reduced the risk of motor impairment in girls but not boys *p* = 0.03 [6]. As nutrition primarily focuses on optimal body composition, it is reasonable to assume that the nutritional requirements of preterm male and female infants may differ due to their distinct body compositions. It is also possible that differences in self-regulated milk volume consumption between male and female term newborns may be responsible for differences in their body compositions. However, no studies in the preterm population have investigated sex differences in nutritional consumption.

Aim: We investigated whether formula intake varies based on infant sex in late preterm infants with ad libitum feeding.

## 2. Methods

This retrospective study was conducted at the University of Mississippi Medical Center with preterm infants (gestational age ≤36 weeks of birth) born between 2015 and 2019 who were fed formula ad libitum before discharge during their primary admissions. Our inclusion and exclusion criteria were as follows:

### 2.1. Inclusion Criteria

- Preterm infants (34 0/7 to 36 6/7 weeks of corrected gestation) were self-regulated, with ad-lib feeding of formula milk during their hospital stay. Corrected gestational age (CGA) is birth gestational age + chronological age in weeks.
- Infants with a hospital stay of at least three or more days after being on ad-lib feeds until the end of the study period, i.e., discharge home.
- Infants free of any respiratory support for at least two days if respiratory support was required initially.

### 2.2. Exclusion Criteria

- Infants were fed infant formula in addition to breast milk or only breast milk. The macro and micronutrient concentrations of the formulas are known, unlike breast milk, whose concentrations vary. As breast milk composition cannot be analyzed, the infants fed with breast milk were, therefore, excluded.
- Infants with a hospital stay less than three days after being on an ad-lib feeding regimen with formula milk were excluded as they did not meet the minimum duration of feeding observation.
- Infants with short gut syndrome, severe chromosomal anomalies, or congenital heart conditions requiring medication.

### 2.3. Nutrition Management and Discharge Criteria of Late Preterm Infants

Our unit policy is to offer oral feeds, either a bottle or breastfeeding, once premature infants are 32 weeks CGA or above. Every infant at 32 weeks and above who is not experiencing increased breathing work and is hemodynamically stable is offered a small volume of 2.5 mL/kg per feed based on feeding cues. Intravenous nutrition is the initial primary mode of nutrition in infants born at <34 weeks GA. Oral feeds are the preferred initial mode of nutrition for infants born at ≥34 weeks unless respiratory support (CPAP or >2 L/min of nasal cannula flow) is needed or infants are medically unstable. Oral feeds are advanced to ad libitum (ad lib for the volume of feeds) once each feed exceeds 75% of the prescribed volume. The feeds are either the mother’s own milk fortified to 22–26 cal/oz depending on weight gain or preterm-transitional formula enriched to 22–26 cal/oz. The choice of milk is based on parental preference. Preterm infants under 34 weeks are offered either their mothers’ breast milk or donor human milk if mothers provide consent. Due to the cost–benefit ratio, preterm infants above 33 weeks CGA are provided with either the mother’s breast milk or formula if the mother cannot provide breast milk. Neonatal nurses feed the infants with formula bottles and record the volume and the type of formula taken in electronic medical records. Infants are observed for a minimum of 3–5 days of consistent oral intake and weight gain before being considered for discharge. Weight was obtained daily before feeding once a day during the night shift. Other discharge criteria to be met included stable thermoregulation at ambient room temperature for at least two days, an apnea-free period of 5–7 days, stable respiratory status without respiratory support for 2–3 days, and an infection-free period.

Information about the type of formula, caloric density, daily oral intake, and feeding duration was obtained from the electronic medical records, and calories and protein were computed from formula nutrient composition tables. Weight percentiles for gestational ages and those appropriate for gestational age were defined based on Fenton’s 2013 growth chart as a weight for gestational age (in grams) between the 10th and 90th percentile.

### 2.4. Sample Size Calculation

There were no previous reports on oral formula consumption in preterm infants. Therefore, we extrapolated our calculations from the data available on older infants’ breast milk consumption study [7]. We estimated the need for 85 males and 85 females (total N = 170) for inclusion in the study after assuming an equal standard deviation of the volume of intake of the formula equal to 14 mL/kg/d, an alpha of 0.05, and an 80% power, to detect a 6 mL/kg/d volume of intake difference between male and female infants (https://clincalc.com/stats/samplesize.aspx) (accessed on 26 January 2024).

### 2.5. Study Period

Entry into the study was when the infant was on more than two days of ad-lib oral feed with formula; the end of the study period was the day of discharge on oral ad-lib feeds.

### 2.6. Data Collected

The data set included the following: demographic data, anthropometric data at birth, at the start of the study, and at the end of the study, maternal data, nutritional intake details such as the mean total volume of intake (mL/kg/d), mean protein intake (g/kg/d) and mean calories (kcal/kg/d) during the study period, and pertinent neonatal morbidity data.

### 2.7. Statistics

An independent sample *t*-test with an a priori significance of <0.05 was used for analyzing continuous variables, and a chi-square test was used for categorical variables.

### 2.8. Institutional Approval

The University of Mississippi Medical Center’s Institutional Review Board approved this retrospective study on 17 May 2021. The IRB File # 2021V0527.

## 3. Results

**Figure 1:**
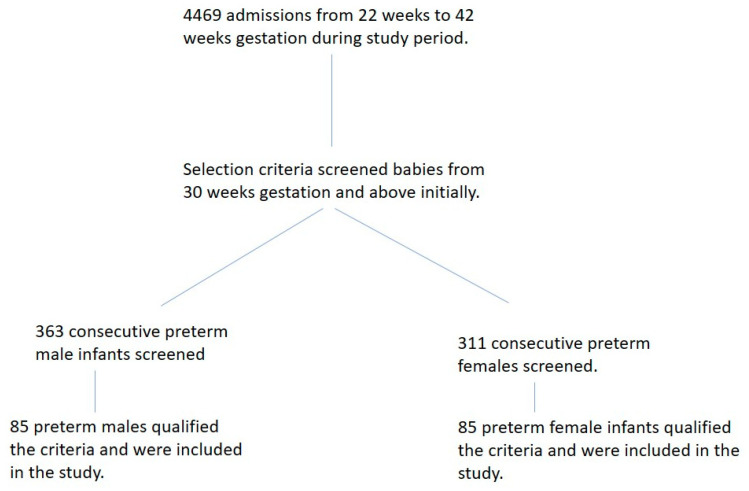
Flow diagram of sample selection.

Four thousand four hundred and sixty-nine infants were admitted to our NICU during the study period. Six hundred and seventy-four preterm infants at 30 weeks and above of gestational age were screened, and 170 qualified the criteria and were included in the study (Figure 1). After satisfying our inclusion and exclusion criteria, we included 85 female and 85 male preterm infants in our study. The mean birth gestational age was 34.0 weeks (sd. ± 0.6 weeks), and the mean birth weight was 2139 g (sd. ± 440 g) for the entire study group. The mean corrected gestational age at the study’s entry was 35.0 weeks (sd. ± 0.6 weeks), and the mean weight at the study entry was 2152.7 g (sd. ± 407 g). There were no sex differences in the birth gestational age or corrected gestational age at the entrance of the study. As expected, female preterm infants were lighter than their similar-age male counterparts at birth (Table 1). However, the weight percentiles at birth, based on sex-specific nomograms, were not different (females 40.5 ± 26.2 percentile vs. males 46.8 ± 27.8 percentile; *p* = 0.13). The duration of observation of oral intake for all infants ranged from 3 to 19 days, with a mean of 6.2 ± 2.4 days.

MORBIDITY: Due to our selection criteria, which included only late preterm infants, none of the study preterm infants had any morbidity, such as Bronchopulmonary Dysplasia (BPD), Necrotizing Enterocolitis (NEC), and Retinopathy of Prematurity (ROP), or hydrocephalus. Of note, there were two instances of Grade 1 intraventricular hemorrhage in male infants and one case of mild ventriculomegaly in a female infant. All infants were discharged home with room air.

This study revealed that most male and female infants were appropriate for gestational age (87% and 90%, respectively), with no significant morbidity. Initially, we analyzed appropriate-for-gestational-age (AGA) infants in our cohort as small-for-gestation infants (SGA) may have different nutritional requirements than AGA infants, and we analyzed AGA separately to exclude SGA as a confounding factor. The analysis of the selected cohort of appropriate gestational age group showed that female infants had a significantly higher mean volume of intake (mL/kg/d), protein intake (g/kg/d), and calorie intake (kcal/kg/d) compared to male infants (Table 1). However, there were no significant differences (*p* = 0.29) found in the mean duration of ad-lib feeding between the sexes: 5.98 days ± 2.81 (mean ± SD) in females versus 6.49 days ± 3.39 (mean ± SD) in males.

Extending the analysis to the entire cohort of infants (including AGA and SGA—small for gestational age) similarly showed that female infants consumed a significantly greater volume per kilogram of body weight than male infants. Female infants consumed 146.3 ± 23.4 mL/kg/d compared to the 136.8 ± 19.8 mL/kg/d consumed by male infants, *p* = 0.005. In addition, females were found to consume significantly more calories (108.7 ± 17.8 kcal/kg/d) than males (101.3 ± 14.4 kcal/kg/d), *p* = 0.003. The protein intake was 3.1 ± 0.5 g/kg/d in females versus 2.94 ± 0.4 g/kg/d in male infants, *p* = 0.03.

As respiratory status influences feeding intake, we also performed an analysis after eliminating infants who required prolonged respiratory support for ≥24 h. Even in this sub-analysis, late preterm female infants consumed a significantly greater volume (female intake was 145.2 mL/kg/d ± 23.4 vs. male intake at 137.0 mL/kg/d ± 19.8) *p* = 0.03, and calories (female = 107.8 kcal/kg/d ± 17.8 vs. male = 102.0 kcal/kg/d ± 14.9) *p* = 0.04, than their male counterparts, thereby confirming the original findings. Though higher in female infants, protein intake was not statistically significant (3.08 ± 0.5 g/kg/d in females vs. 2.96 ± 0.48 g/kg/d in males, *p* = 0.09).

## 4. Discussion

Our research is the first to indicate that there are differences in the consumption of formula milk in late preterm infants based on their sex. We observed that female infants consumed a significantly higher volume and number of calories compared to male infants, particularly those who were appropriate for gestational age (AGA). It is important to note that all infants were allowed to feed freely and control their intake volume during the study period, and most were able to consume orally for over five days. Additionally, none of the infants in the study group had any health issues that could affect their oral intake, so there were no confounding factors related to morbidity.

The issue of variable feed consumption in male and female preterm infants needs to be better understood. A multi-center study across 12 nations evaluated milk intake from 2 weeks to 12 months using a standardized, stable isotope methodology [7]. They observed that boys had significantly higher milk volume consumption than girls (boys–828 mL/d; girls– 772 mL/d; *p* < 0.01). Though this study’s results appear contrary to our findings, it is essential to point out that they did not analyze the intake based on body weight (per kilogram). A similar observation was made in the ‘First-Feed study’, which reported that older male infants consumed a greater volume than females [8]. However, when analyzed per kilogram of body weight, there was no difference in the intake between males and females.

It is worth noting that the two previous studies did not directly observe the actual intake of milk but instead estimated it using isotope-labeled water. Another study that measured infant feed consumption found no difference in volume intake between boys and girls at one month and three months of age [9]. However, when considering volume intake per kilogram of body weight and daily consumption, girls consumed 126 mL/kg/d while boys consumed 114 mL/kg/d. Our study is unique in that it involved the direct measurement and documentation of intake by nurses, and it primarily focused on preterm infants. This sets it apart from earlier studies that do not have these characteristics.

We are unsure why late preterm female infants consume more nutrition than their male counterparts, but we have listed some possible explanations below. One possible explanation is that female preterm infants develop their sucking and swallowing coordination earlier than male infants. This is supported by a prenatal ultrasound study, which demonstrated that females tended to mature their oro-motor skills earlier than males [10].

The period of our study corresponds to a specific period of accelerated growth in female infants. After reviewing the Fenton sex-specific growth curves [11], we found that preterm females had a higher growth velocity than males, especially during the period from 33 to 36 weeks gestation. This growth difference between the sexes is also evident in the WHO’s sex-specific fetal weight nomograms at different gestational ages [12]. From 34 weeks to the term gestation, growth velocities (derived and calculated from original Fenton–2013–and WHO fetal growth charts) is demonstrably higher in female infants than in males (Table 2 and Table 3). Our data also support the fact that late female preterm infants exhibit higher growth velocity. However, this was not statistically significant (*p* = 0.052). There may be a correlation between this relatively higher growth velocity period and higher formula consumption in female late preterm infants.

Short periods of comparatively higher sex-dependent growth have been noted in newborn infants after birth. A Finnish study that evaluated postnatal linear growth velocity between sexes observed that higher linear growth velocity in males in the first 1–2 months of age correlated with a testosterone surge that starts after seven days of age in male infants [13]. Shumei Guo. et al. showed that weight gain was higher among males during the first six months but was later similar to females between six months and two years of age [14]. Additional evidence of a sex-related differential growth rate comes from a study from Hong Kong, which also noted a higher fetal growth rate in females from 28 to 32 weeks gestation [15]. More evidence of variable periods of accelerated growth based on infant sex comes from a population-based birth cohort of the Generation R study, which demonstrated that the estimated fetal weight was higher in males during the first trimester and more elevated in females during the second and third trimesters [16]. Interestingly, the higher third-trimester growth in female infants corresponds to the period of late preterm infants’ 34–36 post-conceptional age, which was our study’s focus. This may explain, to some extent, the higher consumption experienced by late preterm female infants in our study.

Further prospective cohort studies are needed to assess the observed sex differences in preterm infants’ nutritional intake and growth rates.

Sex differences in body composition may present at different post-conceptional ages. Significant fat deposition occurs after 25 weeks of fetal life, in which sex differences may be substantial. Modeled fetal chemical data suggest that the fetus more than doubles its fat mass percentage between 33 and 42 weeks [17]. A body composition study in preterm infants also demonstrated that central subcutaneous adiposity is significantly higher in female preterm infants than in males from 35 weeks gestational age onwards [18]. This study also showed that although subcutaneous fat is relatively higher in female preterm infants from 32 weeks gestation, the difference becomes statistically significant at 38 weeks. Findings from our study suggest that this later gestation period of higher fat deposition in female preterm infants is associated with higher nutritional needs and the volume of intake.

Our initial observations of nutritional intake differences between male and female late preterm infants are preliminary and require further investigation. It is important to note that our study has limitations. We did not examine whether the amount of milk consumed varies with breast milk intake. The mean duration of observation was more than six days. About 39% were observed for 7 to 19 days, which strengthens our study. Our selection criteria allowed us to choose infants who were closer to being healthy, including late preterm babies. We are uncertain whether extremely preterm infants at corrected gestational ages of 34–36 weeks have the same differences in milk intake. However, this also demonstrates the strength of our study, as the absence of significant morbidity among our study infants did not affect our findings related to milk intake.

Our research is important because it highlights the fact that nutritional needs might vary between male and female preterm infants. Therefore, it is crucial to conduct more studies focusing on this aspect and determine whether feeding male and female infants differently could result in better nutritional outcomes. We believe that prospective interventional studies are necessary to understand the specific nutritional requirements of preterm infants, including studies on milk composition, volume intake, and metabolic needs. Moreover, targeted nutritional interventions may be required to maintain the natural sex-specific differences in body composition for optimal health outcomes in premature infants.

## 5. Conclusions

This study observed differences in formula milk consumption between the spontaneous ad libitum feeding of late preterm males and females. The study found that AGA female late preterm infants consumed a greater volume, more protein, and calories than AGA male infants. These findings suggest that sex-specific nutrition studies are also needed in very early preterm infants to understand if there are any sex-specific nutrition requirements.

## Figures and Tables

**Table 1:** Demographic and nutritional characteristics appropriate for gestational age in late preterm infants.

Variable	Females (N = 74)	Males (N = 77)	*p* Value
Gestational age at birth (wk)	34.0 ± 0.69	34.0 ± 0.57	0.48
Birth weight (grams)	2081 ± 308 (−0.11 z)	2342 ± 443 (0.21 z)	0.001
Corrected GA at study (wk)	34.9 ± 0.61	35.1 ± 0.72	0.23
Weight at the start of the study (g)	2079.98 ± 295.6	2350 ± 404.7	0.0001
Duration of observation of intake (d)	5.9 ± 2.8	6.1 ± 3.2	0.69
Mean intake in the study period (mL/kg/d)	145.5 ± 20.8	135.3 ± 19.3	0.002
Mean protein (g/kg/d)	3.07 ± 0.48	2.90 ± 0.45	0.02
Mean calories (kcal/kg/d)	107.8 ± 15.5	100.3 ± 14.4	0.002
Growth velocity in study period(g/kg/d)	8.40 ± 7.66	6.1 ± 6.73	0.052

**Table 2:** Fenton’s sex-based weight-gain nomograms [11].

	Growth Velocity (g/kg/d)		
Gestational Age (Weeks)	Male	Female	Difference
24	19.7	20.7	1
25	19.27	20.17	0.9
26	18.8	19.3	0.5
27	18.88	18.9	0.02
28	19.06	18.82	−0.24
29	19.27	19.09	−0.18
30	19.5	19.3	−0.2
33	16.77	17.24	0.47
34	15.07	15.92	0.85
35	13.35	14.4	1.05
36	11.58	12.84	1.26
37	10.13	10.83	0.7
38	9.27	8.89	−0.38
39	8.8	7.76	−1.04

**Table 3:** WHO fetal sex-based weight-gain nomograms [12].

Growth Velocity Based on WHO Fetal Weights at Different Gestational Ages (g/kg/d)			
Gestational Age (Weeks)	Females	Males	Difference
34	12.82	12.51	0.31
35	11.98	11.65	0.33
36	11.15	10.77	0.38
37	10.34	9.97	0.37
38	9.6	9.2	0.4
39	8.8	8.37	0.43

## Data Availability

The data presented in this study are available on request from the corresponding author. The data are not publicly available due to the need for approval from the University of Mississippi Medical Center IRB.
